# A social network analysis of tourism cooperation in the Yangtze River Delta: A supply and demand perspective

**DOI:** 10.1371/journal.pone.0263411

**Published:** 2022-02-04

**Authors:** Yuewei Wang, Mengmeng Xi, Hang Chen, Xinyang Wu

**Affiliations:** 1 School of Business, Liaoning University, Shenyang, People’s Republic of China; 2 School of Tourism Management, Shenyang Normal University, Shenyang, People’s Republic of China; 3 School of Economics and Management, Northwest University, Xi’an, People’s Republic of China; Northeastern University (Shenyang China), CHINA

## Abstract

This study explores the spatial structure of regional tourism cooperation networks among 27 cities in the Yangtze River Delta from the perspective of supply and demand. Data from the supply network were collected from official news released by the Chinese government and quotations for tour routes published by travel agencies. Travel notes published on tourists’ blog community platforms about their travel experiences were used as source data for the demand network. The degree of cooperation between the cities was analyzed based on the frequency of occurrence and co-occurrence of information on tourist attractions or cities in the Yangtze River Delta region in tourist notes, tourist route quotes, and official news. This study divides 27 cities in the Yangtze River Delta region into three categories: those where supply matches demand (e.g., Shanghai and Nanjing), nine cities where there is a demand lag (e.g., Zhenjiang), and 16 cities where there is a supply lag (e.g., Wuxi). Investigating the differences between the supply and demand networks is helpful to understand the effectiveness of regional tourism cooperation mechanisms and government policies, which is crucial for the sustainability and good governance of regional tourism.

## Introduction

Cooperation is recognized as an important determinant of the competitiveness of tourist destinations [1, 2]. Cities can share tourism resources or innovate tourism products through tourism cooperation [3–5]. In many countries, tourism may be more important than other economic sectors [6]. The development of tourism in China has entered the stage of regional competition and cooperation to maximize the tourist potential of scenic spots, tourist routes, and cities, and it has become increasingly common to seek regional cooperation to promote the sustainable development of tourism [7–9]. Scholars have studied the concept definition, theoretical basis [10, 11], influencing factors and dynamic mechanisms [12], cooperation models, spatial organization [13], evaluated cooperation potential [14], cooperation evolution [15], and other aspects of regional tourism cooperation. Based on the network development trend in regional tourism cooperation [16, 17], some scholars have proposed that tourism destinations are interdependent in regional tourism, which is manifested as groups of nodes and ties representing a certain relationship, and an embedded cooperative structure that constitutes a regional tourism cooperation network [18]. The network view holds that individual and/or organizational behavior is embedded in a network of relational structures and does not act in isolation but in relation to other actors in the network. Based on the concept that network relationship is an organizational resource, cooperative network can be regarded as a process in which several organizations strive to achieve the same goal while controlling their own resources [19]. Therefore, to achieve long-term strategic goals, tourism destinations should not rely on their own efforts, but should actively seek to share information and resources with other organizations in the network, so as to establish a good interaction mechanism [20, 21].

The degree of regional tourism cooperation is closely related to the behavior of governments and tourism enterprises [22]. The policies released by governments and the measures taken by tourism enterprises can affect cooperation and the development of regional tourism [23]. Previous studies have shown that governments and tourism enterprises tend to dominate regional tourism cooperation, especially in co-marketing activities, coordinating tourism resource portfolios, and strengthening formal and informal communication among tourism destinations [24]. Despite great efforts in tourism planning and policymaking, regional government policies are often criticized for being used for political purposes and for implementing tourism policies that are short term in nature and lack comprehensive consideration and coordination [25]. The national government’s policy has also been criticized for not being in line with the needs of tourists. Tourism enterprises may also follow government policies or show a lack of innovation, resulting in the homogenization of tourism products, thus ultimately being unable to meet the needs of tourists. There is growing concern about the effectiveness of policies and initiatives to promote sustainable tourism, as governments and tourism enterprises may not be acting in the interest of tourists. In other words, there may be some contradictions and conflicts between the roles of government and tourism enterprises, and tourists. The behavioral focus of government and tourism enterprises may deviate from tourism demand, which will have a negative impact on the recognition and support for tourists in government policies and the tourism products of tourism enterprises [26]. The sustainable development of tourism should follow the motivation and needs of tourists [27]. The relationship between governments and tourism enterprises and tourists is crucial for maintaining the rationality and effectiveness of policies and initiatives [28]. Government policies and the initiatives of tourism enterprises should ensure that they reflect the wishes of tourists [29]. The government and tourism enterprises should represent the interests of tourists. Government policies and the initiatives of tourism enterprises are ineffective without the approval and support of tourists [30].

Tourists’ views on regional tourism cooperation may have a significant impact on the formulation of appropriate tourism policies and products. Investigating the differences between tourists’ demands and government policies (initiatives of tourism enterprises) is helpful for fully exploring the effectiveness of tourism cooperation mechanisms and government policies (initiatives of tourism enterprises). However, few scholars have paid attention to the differences between tourist demand and government (tourism enterprise) approaches in the cooperation of tourist destinations. Researchers and policymakers fail to take into account the importance of harmonizing government policies (tourism enterprise initiatives) with tourists’ perceptions of destination cooperation [31]. Tourists are the perceivers of regional tourism cooperation, who can connect different tourist destinations through their freely chosen spatial behaviors and movements. Gozzo and Tomaselli studied regional tourism cooperation formed by tourist flows, and confirmed that tourist flows influence the form, scale, and structure of cooperation between different tourist destinations [32]. The spatial behavior of tourists is potentially important in understanding regional tourism cooperation [33], but has received little attention in existing studies. From the perspective of tourist demand, regional tourism cooperation analysis is inclined to investigate the cooperative relationship between tourist behavior and spatial movement in destinations [33]. Many scholars use different theories to interpret tourists’ attitude toward the development of tourism. Researchers surveyed tourists on their perceptions of the costs and benefits of travel, as well as their attitudes toward government policies and initiatives by tourism enterprises [34]. Policymakers would benefit from a better understanding of tourists’ attitudes toward government policies and the development of local tourism; however, only a few studies have analyzed tourist perceptions and/or attitudes toward destination collaboration during the planning process [35]. Therefore, one of the aims of this study is to help governments and tourism enterprises understand the impact of tourists’ spatial behavior on tourism destination cooperation.

In recent years, there has been a growing trend of using the Internet as the main data source for web text analysis in tourism literature. Current applications and configurations are based on a good theoretical basis and sound methods [36], as a result, web text analysis has been increasingly applied to different disciplines, including tourism and hospitality, especially in terms of tourism supply, destinations, policy systems, tourist movements, and behavioral patterns. With the development of the Internet, an increasing number of travelers are using online travel notes to share or find information and to make travel decisions. Travel notes record the perceptual memory of tourists and record the travel routes that reflect the cooperation and interaction between tourist destinations from the perspective of demand. The government and tourism enterprises also use websites and Internet platforms to release official news and tourism route information, and to put forward policies or measures to promote cooperation among tourism destinations, improve the popularity of tourist destinations, and increase the possibility of tourists visiting specific tourist destinations. The travel notes released by tourists and the web texts published by governments (tourism enterprises) both contain relevant information on regional tourism cooperation. As a result, they are an important data source for the study of regional tourism cooperation. Consistency between tourists’ perceived memories and government policies (tourism enterprises’ measures) is essential for sustainability and good governance in the tourism industry. From a practical point of view, the tourist and government’s (tourism enterprises) perspective of tourism destination cooperation are closely related. A better understanding of tourism destination cooperation from the perspective of tourist needs will help to strengthen decision-making by governments (tourism enterprises). Therefore, the main purpose of this study is to compare two different perspectives of tourism destination cooperation: the perspective of governments and tourism enterprises (supply perspective) versus the perspective of tourists (demand perspective). To do so, this study first describes the supply network at a government (tourism enterprise) level and the demand network at a tourist level based on the social network analysis method; second, it compares the similarities and differences between the supply network and the demand network in terms of the rights centrality of nodes in the overall structure, and creates an explanatory analysis of these differences; and third, it explores the methods of cooperative research on tourism destinations based on web texts, and provides practical applications for the formulation of effective policies and measures.

## Research data and methods

### Analysis framework of the regional tourism cooperation network

Theoretically, the main supply body of regional tourism cooperation should include governments, tourism enterprises, and non-governmental tourism organizations, but in the absence of non-governmental tourism organizations in China, governments and tourism enterprises become the core body of regional tourism cooperation supply networks. On the demand side, the multi-destination travel behaviors of tourists contribute to regional tourism cooperation demand networks. In addition, government regulatory power, tourism enterprise executive power, and the market are all important driving forces for the formation of regional tourism cooperation networks. The government’s regulatory power and tourism enterprises’ executive power promote the orderly development of optimal destination combinations, and constitute the artificial external driving force. They play a role in promoting, delaying, or revising the formation and evolution of regional tourism cooperation supply networks. Specifically, governments play a leading role in regional tourism cooperation by formulating regional tourism cooperation plans to improve relevant infrastructure and to enhance the image of regional tourism. In response, tourism enterprises jointly design tourism routes, carry out publicity and promotional activities, provide reception services and other measures, and jointly build the supply network for regional tourism cooperation.

**Fig 1:**
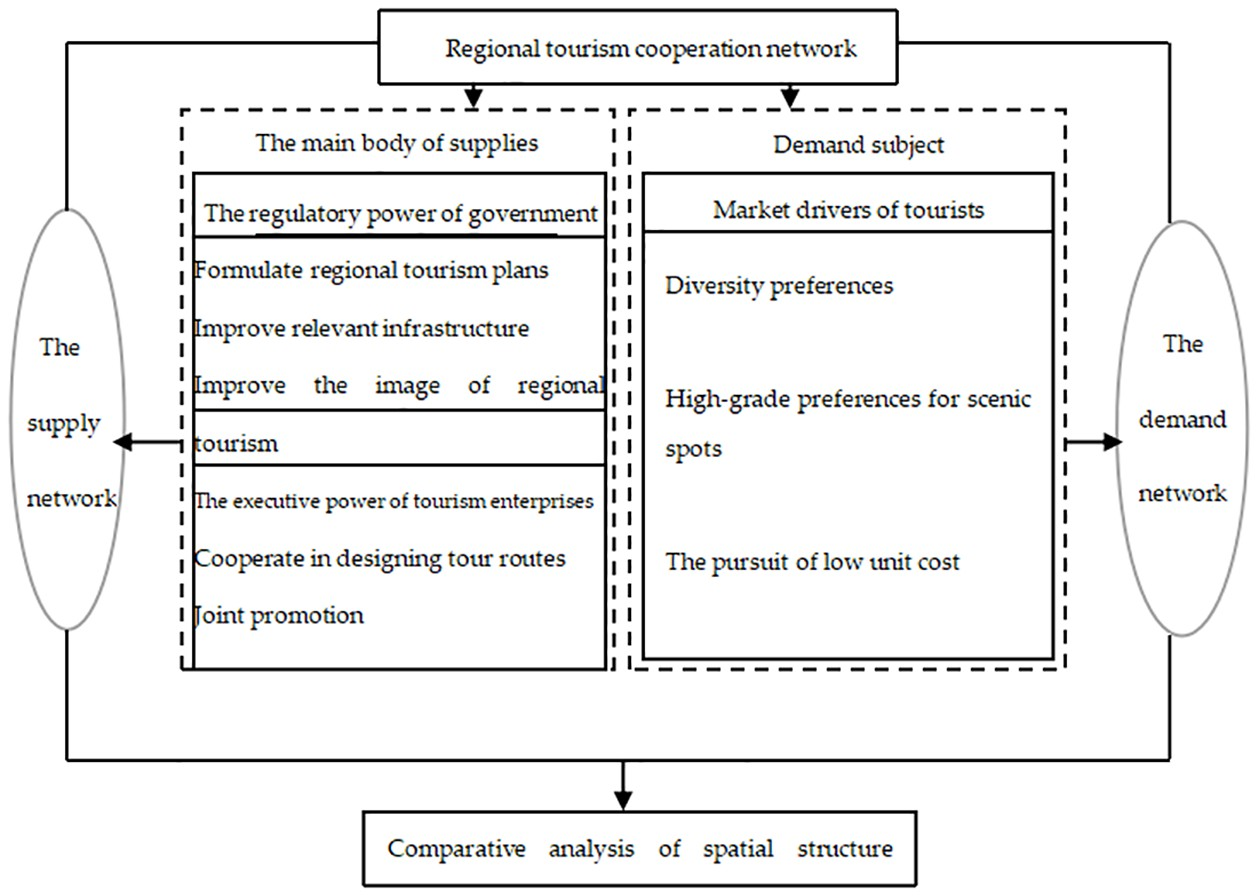
Analysis framework of a regional tourism cooperation network.

The demand characteristics of tourists—based on their diverse preferences, such as a preference for exceptional scenic spots and low-cost travel—drive market forces. As these preferences are a type of expansion from the inside to out, they can be regarded as the internal driving force. Specifically, tourists’ multi-destination travel behaviors objectively contribute to the formation of tourism cooperation demand networks between different regions on the demand side. Whether government policies and tourism enterprise measures effectively meet the needs of tourists is reflected in the matching relationship between the supply and demand networks. Under the joint action of external and internal driving forces, when the supply and demand networks are well matched, they will greatly promote the development of regional tourism cooperation networks. However, the formation and evolution of regional tourism cooperation networks will be delayed if the development of the supply and demand networks deviate from each other (Fig 1).

### Study area

The Yangtze River Delta is located in the lower reaches of the Yangtze River in China, close to the Yellow Sea and the East China Sea, and at the intersection of the river and sea. The Yangtze River Delta is China’s regional economic center.

The Yangtze River Delta is one of the most mature tourism regions in China, and its tourism resources are extremely rich and have strong complementarity. As of December 2019, a total of 1,240 National A-level tourist attractions in China are located in the Yangtze River Delta region, including 53 5A level scenic spots, 469 4A level scenic spots, 488 3A level scenic spots, 227 2A level scenic spots, and three 1A level scenic spots. For example, Shanghai has natural tourism resources such as Sheshan, Chongming Island, Huangpu River, Dongtan Bird Nature Reserve, and Gulf National Forest Park, as well as cultural tourism resources such as Yu Garden, Zhujiajiao, the site of the First Congress of the Communist Party of China and the former residence of Sun Yat-sen. There are three world cultural heritage sites and eight intangible cultural heritage sites in Jiangsu Province, as well as a large number of scenic spots, cultural relics protection units, nature reserves, and forest parks. There are 17 5A-level scenic spots in Zhejiang province, including the famous West Lake scenic spot. In addition, Zhoushan, China’s largest archipelago, and Qiandao Lake, known as the world’s most beautiful waterbody, are also located in Zhejiang Province. In addition to the landscape scenery, Wuzhen, Xitang, Nanxun ancient town, and other famous ancient water towns are also located in Zhejiang Province. As a famous province of tourism resources in China, Anhui province boasts beautiful natural scenery. For example, the famous Huangshan Mountain Scenic Area is located in Huangshan City of Anhui Province, and Jiuhua Mountain Scenic Area is located in Chizhou city of Anhui Province. At present, there are 600 A-level and above tourist attractions located in Anhui Province.

The tourism industry in the Yangtze River Delta region plays an important role in the development of China’s tourism industry. According to the data released by the 2019 Yangtze River Delta Eco-Tourism Integration Summit forum, the Yangtze River Delta received about 30 million inbound tourists in 2018. The number of 5A scenic spots in the Yangtze River Delta accounts for more than 20% of China’s total, and the number of star-rated hotels accounts for more than 16% of China’s total. Companies from the Yangtze River Delta region accounted for more than half of the top 20 list of China Tourism Group in 2018. Therefore, the discussion of regional tourism cooperation in this region has strong reference value. The Yangtze River Delta Tourism City Summit has been held since 2003; China has also signed a regional tourism cooperation agreement to advocate the construction of a barrier-free tourism zone. Twenty-seven cities in the Yangtze River Delta region have participated in this cooperation agreement since 2019.

Specifically, the 27 cities include Shanghai city, Nanjing, Wuxi, Changzhou, Suzhou, Nantong, Yangzhou, Zhenjiang, Yancheng, and Taizhou in Jiangsu province; Hangzhou, Ningbo, Wenzhou, Huzhou, Jiaxing, Shaoxing, Jinhua, Zhoushan, and Taizhou in Zhejiang Province; and Hefei, Wuhu, Ma’anshan, Tongling, Anqing, Chuzhou, Chizhou, and Xuancheng in Anhui Province. In this study, tourism destination cooperation in the Yangtze River Delta region refers to the cooperation and interaction between these cities through the interaction of the tourism market, the sharing of information, and the creation of products and brands, among others, to achieve a mutual development goal.

### Data collection and analysis

In this study, Python crawler is used to collect web text data. The basic idea of Python web crawler is as follows: first, get the source code of the web page (right mouse click > display the source file); second, find the information matching the requirements in the source code of these web pages and connect to the database; third, each successfully matched information is stored in the database until all web pages have been retrieved. This study mainly focuses on the travel notes published by tourists in online communities, the official news published by the Chinese government on government websites, and the tour routes published by travel agencies on their websites. These datasets collection methods are in line with the terms and conditions of the above websites. Based on accessibility, trustworthiness, and online user-generated content (UGC) that is readily available and free of charge, tourism cooperation is analyzed from the perspective of demand and supply. The use of multiple data sources allows for mutual support, thus improving the credibility and reliability of research results [37]. Some researchers have used evidence from a variety of sources in their studies, including government documents, non-governmental publications, and interviews [18]. Ctrip is China’s leading online travel service company, providing more than 90 million members with hotel reservation, hotel review and special offer hotel query, air ticket reservation, air ticket query, schedule, ticket query, flight query, vacation reservation, business travel management, and other services. Ctrip community records tourist attractions in hundreds of thousands of cities around the world. Tourists can not only access free travel guides, self-help Tours, budget Tours, self-driving Tours, and other complete travel guides, as well as other tourists’ travel diaries, but also upload travel notes on these platforms. Mafengwo is a tourism social networking site, a data-driven platform, and a new type of tourism e-commerce. It provides transportation, hotels, attractions, catering, shopping, cars, local entertainment, and other information and product booking services for 60,000 tourist destinations worldwide. Since the operation of Mafengwo travel network in 2010, the information content of Mafengwo community has been continuously enriched and improved by a large number of travelers sharing independently, and it has become the first choice of the young generation of "tourism treasures." Other major OTA platforms include Tuniu.com and Sina.com. Tuniu.com was founded in 2006 with the mission of making travel easier. Founded in Nanjing, Tuniu was successfully listed on NASDAQ on May 9, 2014. As a leading leisure travel company in China, Tuniu provides online and offline consumers with self-guided cruise scenic spot tickets and company travel tickets, hotels, and other products and services. Sina.com, China’s largest Chinese language web portal, was founded in Beijing in 1998. It has more than 500 million users and receives millions of blogs or comments every day. These four blogs, or comment sites, allow Internet users to access UGC from the Internet via mobile devices and to share their interests with friends and family.

A total of 123,011 travel notes, referred to as tourist texts, were obtained. Among them, we deleted travel notes that only involved one city and the promotional texts of businesses, and finally obtained 106,764 valid travel notes. Second, 558 official policy documents and news items on tourism cooperation between the 27 cities in the Yangtze River Delta that were published by government departments on official websites, referred to as government texts, were collected. After deleting the texts involving only one city, 437 valid texts were obtained. Third, a total of 8,132 tourist routes in the Yangtze River Delta were obtained from the quotation lists of the top 100 travel agencies and mainstream OTA platforms, such as Ctrip, and are referred to as tourism enterprise texts. After deleting the routes involving only one city, 7,005 valid routes were obtained. As the number of travel notes in 2020 would have been affected by the COVID-19 epidemic, the study covers the period from January to December 2019.

The first step was to process the tourist texts and tourism enterprise texts into directed city relationship data. A scenic spot dictionary was built for each city. Using the scenic spot dictionary, we identified the tourists in the Yangtze River Delta based on the direction of the travel routes, such as Shanghai→Suzhou→Hangzhou→Nanjing. Tourism enterprise texts were processed in the same way. The second step was to process government texts as directed partnership data. For example, when Nanjing initiated and held the Nanjing Metropolitan Circle Culture and Tourism Cooperation Joint Conference, Nanjing, Huai’an, Yangzhou, Zhenjiang, Chuzhou, Ma’anshan, Wuhu, and Xuancheng—the eight leading cities—attended the conference. The data for oriented cooperation were Nanjing→Huai’an, Nanjing→Yangzhou, Nanjing→Zhenjiang, Nanjing→Chuzhou, Nanjing→Ma’anshan, Nanjing→Wuhu, Nanjing→Xuancheng. The directed data of tourist texts, tourism enterprise texts, and government texts were sorted into EXCEL tables and then transformed into a directed matrix using Python software.

The row of the matrix represents the starting point, the column represents the end point, and both the starting and end points are composed of the 27 cities in the Yangtze River Delta. If the directed data of A→B are α, and the directed data of B→A are β, then the total directed data of A and B are α+β. Thus, three multivalued adjacencies with 27 rows and 27 columns are established. The directed matrix formed by tourism enterprise texts and government texts is categorized as the supply network matrix and that formed by tourist texts as the demand network matrix. Finally, a binary matrix is established by repeated testing to select the appropriate endpoint value.

### Analysis methods

#### Social network analysis

Social network analysis has been adopted in new economic sociology based on social measurement to examine the relationship between social networks and actors [32]. It includes an overall structure analysis and an individual analysis. The overall structure index of the tourism network includes network density, core–edge, and so on. Individual analysis can be measured using the centrality of power. Centrality is an important concept in social network analysis. It is often used to analyze and reflect the differences between individual actors in terms of rank and advantage, and often regarded as the difference of rights. Social network analysis makes a quantitative study of rights from the perspective of relationships, and provides various forms of quantitative centrality indexes, such as degree centrality, closeness centrality, and betweenness centrality, to evaluate the superiority of the importance status (level) of individuals in a network (Table 1).

**Table 1:** Tourism network structure evaluation indicators and their meanings.

	First grade indicators	Second grade indicators	Index significance
Overall evaluation	Network density	—	The ratio of the actual number of relationships to the theoretical maximum number of relationships
	Core–edge model	—	The location of a node in a network
Individual evaluation	Centrality of power	Betweenness centrality	Node mediation
		Degree centrality	Degree of node importance
		Closeness centrality	Node connectivity

#### Entropy method

The entropy method determines the weight of each index according to the degree of variation in the index value. Compared with Analytic Hierarchy Process (AHP) and Delphi, the entropy method is more reliable and widely used in comprehensive multi-index evaluations. The detailed calculation steps of the entropy method are as follows.

Step 1: Extreme value standardization
$$\text{Positive}\;\text{dimensions}:{\text{A}}_{\text{ij}}=\frac{{\text{X}}_{\text{ij}}-\text{min}({\text{X}}_{\text{j}})}{\text{max}({\text{X}}_{\text{j}})-\text{min}({\text{X}}_{\text{j}})}$$ (1)
$$\text{Negative}\;\text{dimensions}:{\text{A}}_{ij}^{\prime }=\frac{\text{max}({\text{X}}_{\text{j}})-{\text{X}}_{\text{ij}}}{\text{max}({\text{X}}_{\text{j}})-\text{min}({\text{X}}_{\text{j}})}$$ (2)
  In formulas (1) and (2), A_ij_ represents the *j*th indicator value of the *i*th system, X_ij_ and represents the original value of the *j*th index of the *i*th system.
Step 2: Entropy method:
$${\text{Y}}_{\text{ij}}=\frac{{\text{A}}_{\text{ij}}}{\sum_{\text{i}=\text{t}}^{\text{m}}{\text{A}}_{\text{ij}}},$$ (3)
$${\text{E}}_{\text{j}}=-\text{K}\sum_{\text{i}=1}^{\text{m}}\left({\text{Y}}_{\text{ij}}\times \text{ln}{\text{Y}}_{\text{ij}}\right)\;\text{K}=\frac{1}{\text{ln}\;\text{m}},0\leq {\text{E}}_{\text{j}}\leq 1,$$ (4)
$${\text{D}}_{\text{j}}=1-{\text{E}}_{\text{j}},{\text{W}}_{\text{i}}=\frac{{\text{D}}_{\text{j}}}{\sum_{\text{j}=1}^{\text{n}}{\text{D}}_{j}},$$ (5)
  and
$${\text{S}}_{\text{ij}}={\text{W}}_{\text{i}}\times {\text{A}}_{\text{ij}},{\text{S}}_{\text{i}}=\sum_{\text{i}=1}^{\text{n}}{\text{S}}_{\text{ij}}$$ (6)
  In formulas (3)–(6), Y_ij_ is the disorder value of the index, E_j_ is the information entropy of the index, D_j_ is the redundancy of index information entropy, W_i_ indicates the weight of an indicator, and S_ij_ is the evaluation score of a single indicator.

## Result analysis

### Analysis of spatial structure characteristics of the regional tourism cooperation network in the Yangtze River Delta

#### Construction of the supply and demand networks

**Fig 2:**
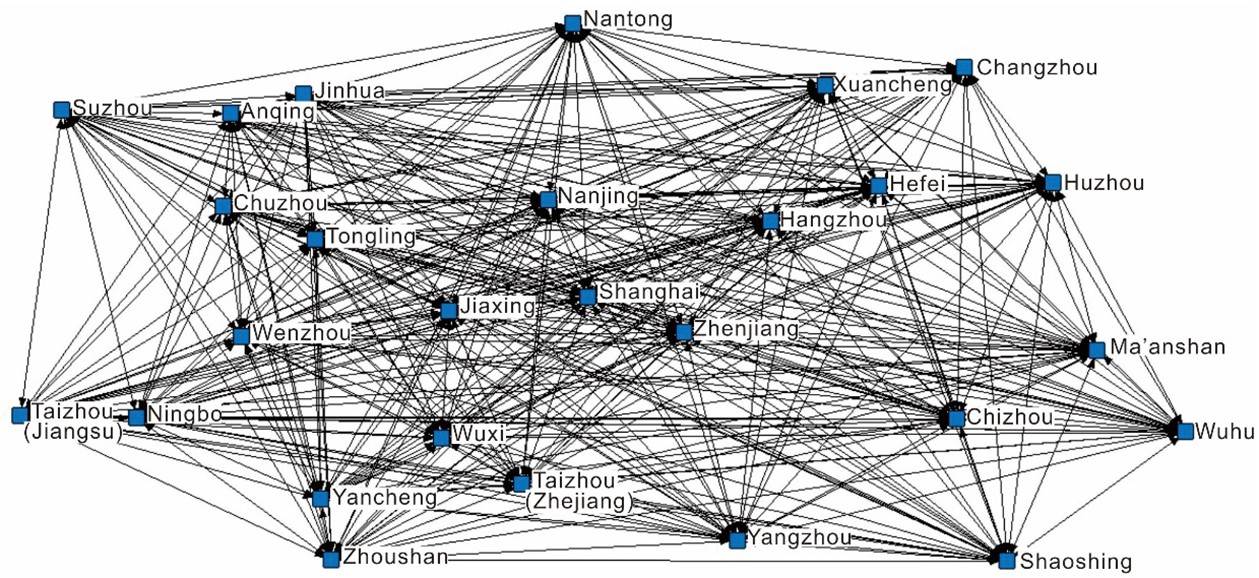
The supply network of regional tourism cooperation in the Yangtze River Delta.

**Fig 3:**
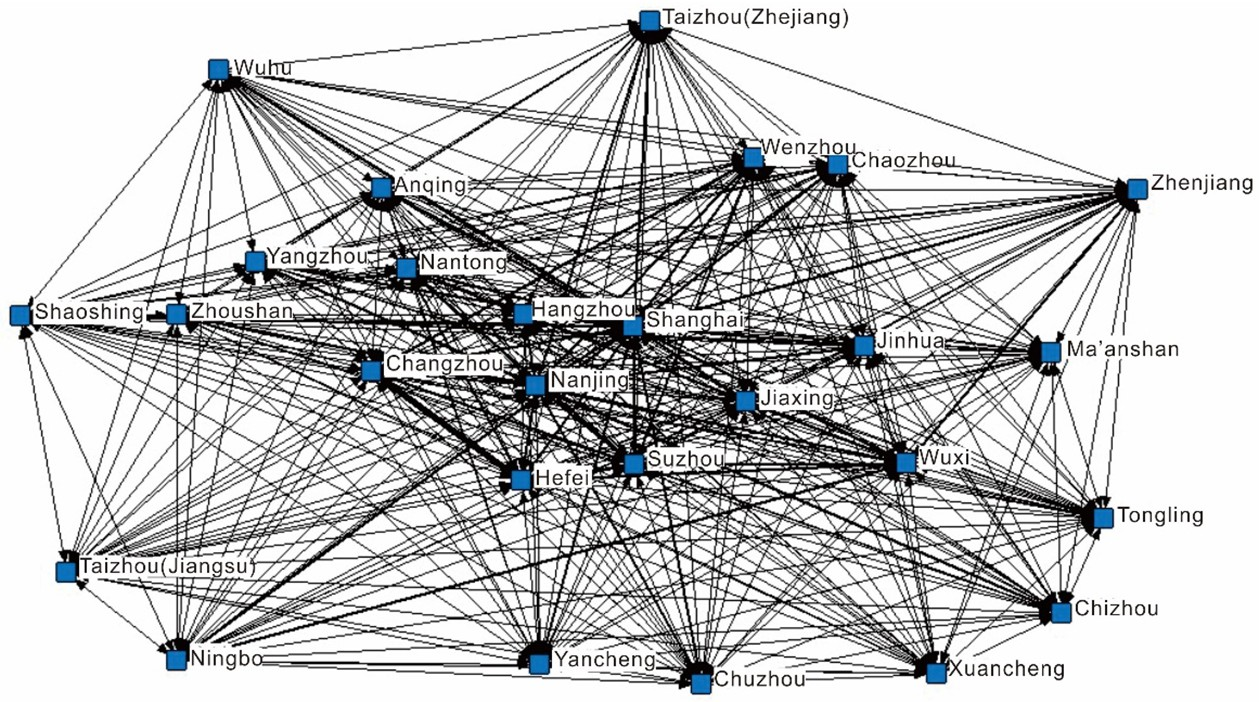
The demand network of regional tourism cooperation in the Yangtze River Delta.

This study uses Ucinet6.0 software to build the supply (Fig 2) and demand networks (Fig 3) for regional tourism cooperation in the Yangtze River Delta. By comparing the two, similarities and differences between the supply and demand networks can be found. Overall, the two networks cover all 27 cities in the Yangtze River Delta and have no isolated points. The network densities of the supply and demand networks are 0.56 and 0.6, respectively, and the number distribution of network connections is 232 and 288, respectively, indicating that cities in the Yangtze River Delta have carried out extensive tourism cooperation at both the supply and demand levels. The distribution of lines in the two networks is very uneven, with fewer bold lines and more fine lines. This indicates that in terms of the intensity of cooperation, only a few cities carried out frequent tourism cooperation initiatives, while most cities had relatively few tourism cooperation initiatives.

#### Analysis of core–edge structure characteristics

According to the analysis of the core–edge structure (Table 2), there are eight node cities in the demand network that are core members of the network, and only four in the supply network. In the supply network, Shanghai, Nanjing, Suzhou, and Hangzhou have been the focus of government and tourism enterprises for a long time. Many regional tourism cooperation initiatives in the Yangtze River Delta include these cities. The encryption and optimization of transportation networks in the Yangtze River Delta, including airports (e.g., Hongqiao airport, Shuofang airport, and Putuo Mountain airport), high-speed railways (e.g., Ningbo-Hangzhou high-speed railway and Shanghai-Ningbo high-speed railway), and expressways (e.g., Shanghai-Hangzhou high-speed expressway), provide efficient and diverse autonomous transportation options for tourists for cross-city travel in the Yangtze River Delta region, to meet tourists’ preference for low unit costs and high-grade scenic spots. In addition to Shanghai, Nanjing, Suzhou, and Hangzhou, cities such as Wuxi, Nantong, Ningbo, and Hefei have also become core nodes in the demand network. Therefore, government and tourism enterprises need to pay more attention to these cities in the future.

**Table 2:** Core–edge structure of the demand and supply networks.

Province/city	The demand network		The supply network	
	Core area	Edge area	Core area	Edge area
Shanghai	Shanghai		Shanghai	
Jiangsu	Nanjing, Suzhou, Wuxi, Nantong	Yangzhou, Zhenjiang, Taizhou, Changzhou, Yancheng	Nanjing, Suzhou	Wuxi, Changzhou, Nantong, Yancheng, Yangzhou, Zhenjiang, Taizhou
Zhejiang	Hangzhou, Ningbo	Jiaxing, Huzhou, Shaoshing, Jinhua, Zhoushan, Taizhou, Wenzhou	Hangzhou	Ningbo, Jiaxing, Huzhou, Shaoshing, Jinhua, Zhoushan, Taizhou, Wenzhou
Anhui	Hefei	Anqing, Chizhou, Xuancheng, Tongling, Chuzhou, Ma’anshan, Wuhu		Hefei, Wuhu, Ma’anshan, Chizhou, Xuancheng, Tongling, Anqing, Chuzhou

#### Analysis of rights centrality in network nodes

This study uses Ucinet6.0 software to calculate the centrality index of nodes in the supply and demand networks.

**Fig 4:**
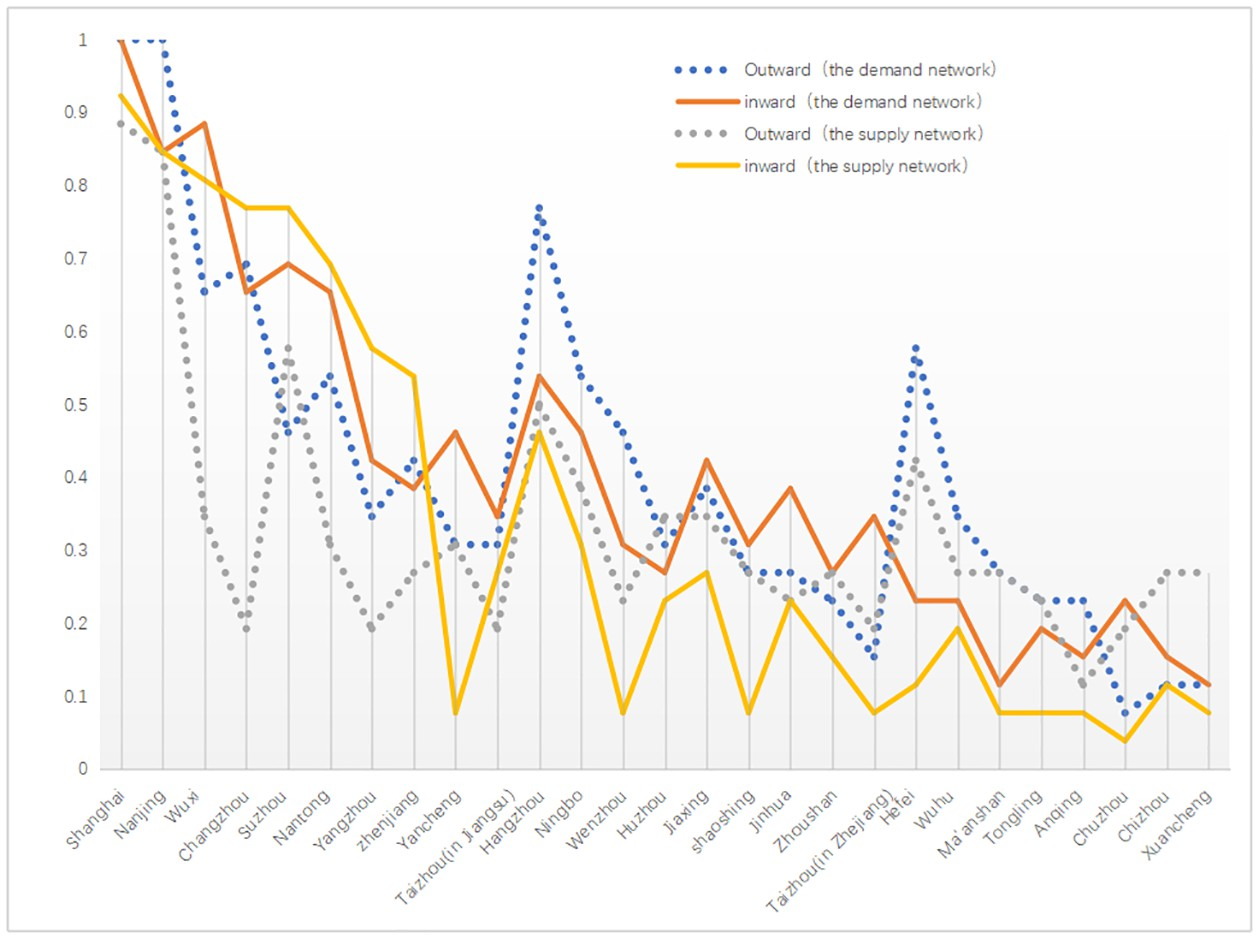
The degree centrality indexes of supply and demand networks in regional tourism cooperation.

In the demand network of regional tourism cooperation in the Yangtze River Delta, based on actual tourism flow, Shanghai and Nanjing’s inward and outward degree centrality are both over 0.7, far higher than the figures for other cities in the region (Fig 4). They are the undisputed distribution centers in the regional tourism cooperation network in the Yangtze River Delta. Wuxi, Changzhou, Suzhou, Nantong, Hangzhou, and Ningbo are secondary distribution centers, with their inward and outward degree centrality above 0.4. Among them, the inward degree centrality of Wuxi is much higher than its outward degree of centrality, indicating that it is a prime tourist destination in the Yangtze River Delta. The outward centrality of Hangzhou is much higher than its inward centrality, indicating that Hangzhou is an important diffusion center for tourists in the Yangtze River Delta region. In the case of the following 12 cities, Yangzhou, Zhenjiang, Yancheng, Taizhou (in Jiangsu province), Wenzhou, Huzhou, Jiaxing, Shaoshing, Jinhua, Zhoushan, Hefei, and Wuhu, their inward and outward centrality degrees are relatively small, between 0.2 and 0.4, respectively. The outward centrality of Hefei is much higher than its inward centrality, which indicates that Hefei plays an important role as a diffusion center in Anhui Province. The inward and outward degree of centrality of the following seven cities, Taizhou (in Zhejiang province), Ma ’anshan, Tongling, Anqing, Chuzhou, Chizhou, and Xuancheng, are all less than 0.2, which makes them edge regions in the regional tourism cooperation network of the Yangtze River Delta.

From the comparative analysis of the supply and demand networks, there are eight distribution centers and sub-distribution centers in the demand network, and only four in the supply network. This indicates that in addition to Shanghai, Nanjing, Suzhou, and Hangzhou, the governments and tourism enterprises should focus more on the distribution function of the four nodes of the tourism flow in Wuxi, Changzhou, Nantong, and Ningbo. In the supply network, the outward centrality of Yangzhou, Zhenjiang, Hangzhou, Wenzhou, and Hefei is much lower than its value in the demand network, which indicates that the governments and tourism enterprises have not fully understood the diffusion effect of these cities in the Yangtze River Delta regional tourism cooperation network. In the supply network, the inward degree centrality of Yancheng, Wenzhou, Shaoshing, Taizhou (in Zhejiang province), Hefei, and Tongling is much lower than its value in the demand network, indicating that these cities possess strong tourist attractions, and hence governments and tourism enterprises need to pay more attention to them.

**Fig 5:**
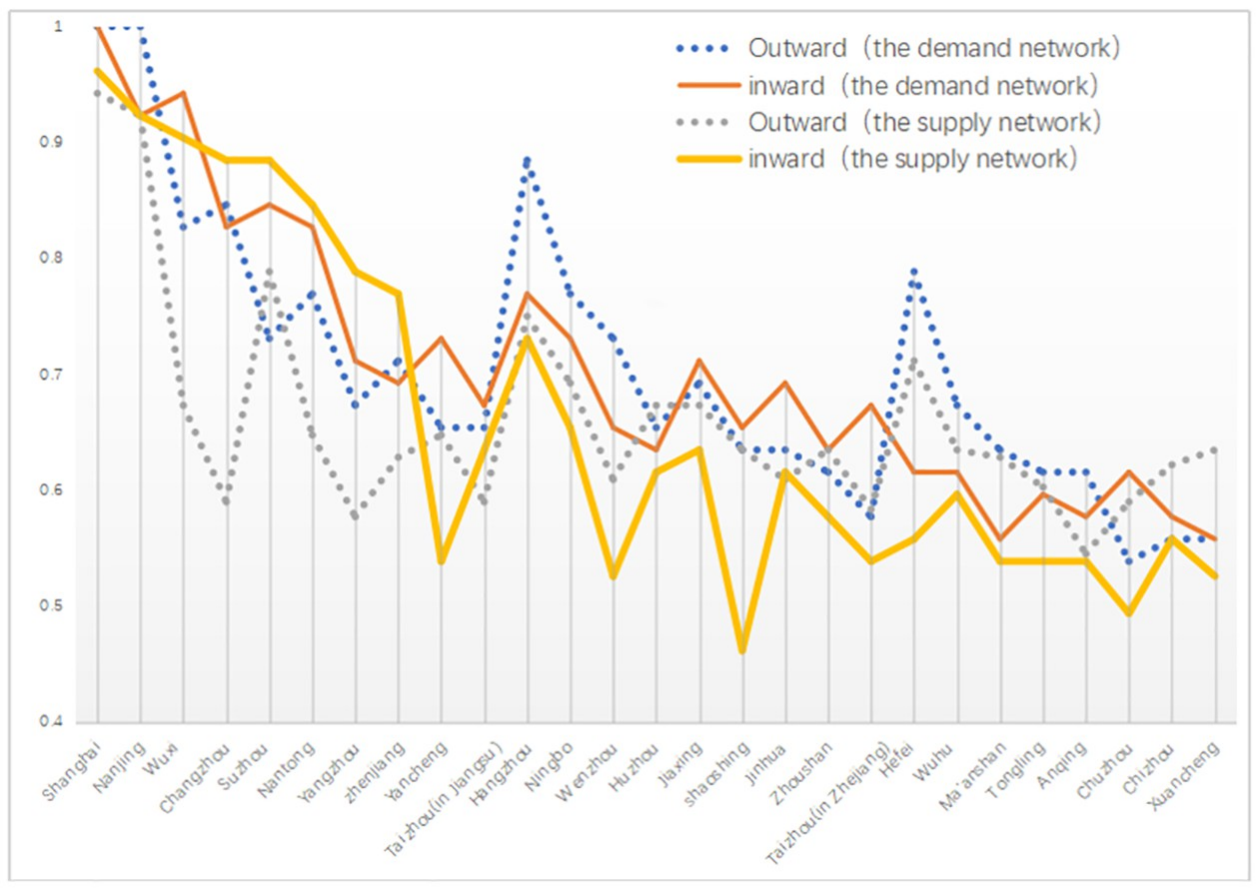
The closeness centrality indexes of supply and demand networks in regional tourism cooperation.

As can be seen from Fig 5, the connectivity of the regional tourism cooperation network in the Yangtze River Delta is relatively good in both the demand and supply networks, and there are no isolated nodes. This may be because the Yangtze River Delta region has formed an efficient and fast transportation network, providing tourists in the Yangtze River Delta region with efficient and diverse travel options, and greatly improving connectivity within the regional tourism cooperation networks in the Yangtze River Delta.

In both the supply and demand networks, the centrality of Shanghai and Nanjing is much higher than that of other regions, indicating that these two cities have considerable control over other regions in the Yangtze River Delta regional tourism cooperation network, and are in a core hub position.

**Fig 6:**
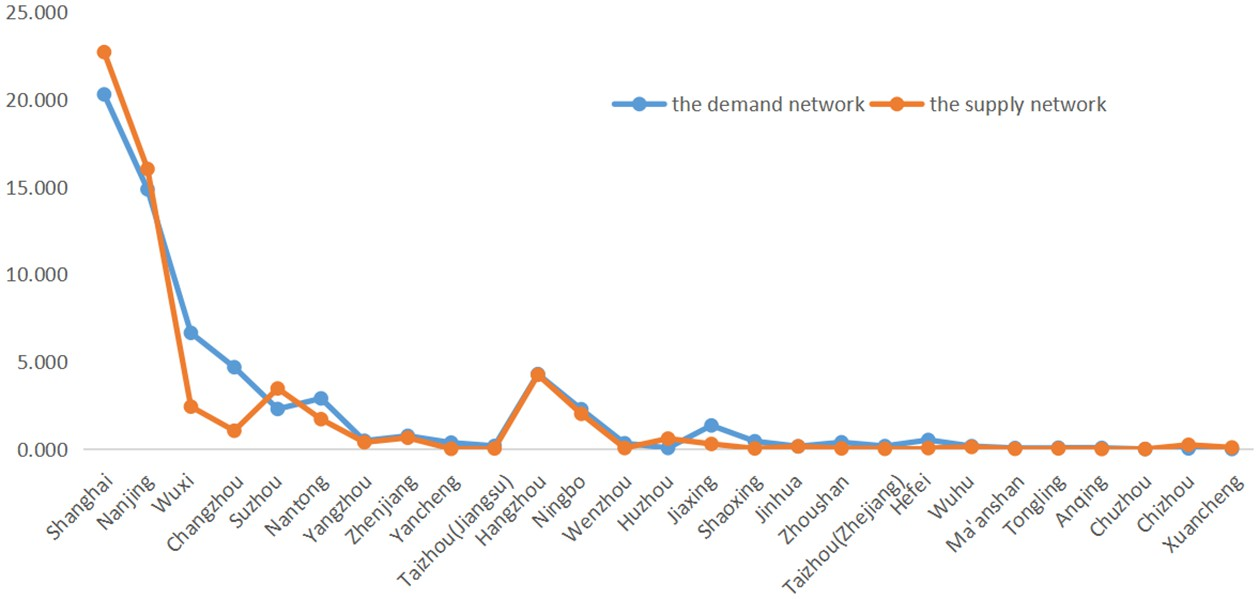
The betweenness centrality index of the demand and supply networks.

The betweenness centrality index values of seven cities, Wuxi, Changzhou, Suzhou, Nantong, Hangzhou, Ningbo, and Jiaxing, are also greater than 1 (Fig 6), indicating that they are important transit nodes for tourism in the Yangtze River Delta region. The index values of betweenness centrality of 11 cities, Yangzhou, Zhenjiang, Yancheng, Taizhou (in Jiangsu province), Wenzhou, Shaoshing, Jinhua, Zhoushan, Taizhou (in Jiangsu province), Hefei, and Wuhu, are between 0 and 1, indicating that their transfer effect on tourism flow is not strong. The centrality of seven cities, Chaozhou, Ma’anshan, Tongling, Anqing, Chuzhou, Chizhou, and Xuancheng, is basically 0, indicating that they have no control over other nodes and are not on the shortest route to any two tourist destinations. From the comparative analysis of the supply and demand networks, it is clear that government and tourism enterprises need to pay more attention to the transfer roles of Wuxi, Changzhou, Nantong, and Jiaxing.

### Analysis of the difference between the supply and demand networks

Based on the entropy method, this study calculates the comprehensive evaluation value of the right centrality index of nodes in the supply and demand networks to analyze the differences in the supply and demand networks associated with regional tourism cooperation in the Yangtze River Delta.

According to Table 3, only Shanghai and Nanjing have roughly similar indexes of centrality of nodes in the supply and demand networks. Suzhou, Zhenjiang, Yangzhou, Jinhua, Huzhou, Wuhu, Chizhou, Xuancheng, and Chuzhou show a type of demand lag. Although these cities are valued and supported by governments and tourism enterprises, their ability to attract tourism flow is small, and their role as central nodes in the demand network is weaker than that in the supply network, probably due to the lack of tourism resources or being over-shadowed by other well-known scenic spots. Wuxi, Changzhou, Hangzhou, Nantong, Ningbo, Jiaxing, Hefei, Yancheng, Wenzhou, Zhoushan, Taizhou (in Jiangsu province), Taizhou (in Zhejiang province), Ma’anshan, Shaoshing, Tongling, and Anqing show a type of supply lag. Unique tourism resources, product differentiation, and the proximity of famous scenic spots, strengthen relations between regional tourism authorities and catering for tourist preferences for high value and low costs, thus attracting more tourism flow in the regional tourism cooperation network. This plays a more important role than governments and tourism enterprises.

**Table 3:** Centrality index of nodes in the demand network and the supply network.

Province/city		The demand network			The supply network		
		Betweenness centrality	Degree centrality	Closeness centrality	Betweenness centrality	Degree centrality	Closeness centrality
Shanghai	Shanghai	131.8	26	1	199.5	23.5	0.913
Jiangsu	Nanjing	96.54	24	0.934	149.6	22	0.867
	Wuxi	43.16	20	0.82	15.75	15	0.722
	Changzhou	30.41	17.5	0.754	6.79	12.5	0.678
	Suzhou	14.88	15	0.708	48.51	17.5	0.758
	Nantong	18.82	15.5	0.713	11.10	13	0.672
	Yangzhou	3.069	10	0.620	2.51	10	0.612
	Zhenjiang	4.896	10.5	0.627	4.19	10.5	0.625
	Yancheng	2.372	10	0.621	0.07	5	0.549
	Taizhou	1.136	8.5	0.598	0.20	6	0.560
Zhejiang	Hangzhou	27.85	17	0.749	27.59	12.5	0.659
	Ningbo	14.79	13	0.667	13.08	9	0.605
	Wenzhou	2.501	10	0.621	0.36	4	0.527
	Huzhou	0.386	7.5	0.585	3.88	7.5	0.585
	Jiaxing	8.763	10.5	0.627	1.88	8	0.592
	Shaoshing	2.888	7.5	0.585	0.20	4.5	0.499
	Jinhua	0.601	8.5	0.599	0.98	6	0.559
	Zhoushan	2.130	6.5	0.572	0.22	5.5	0.560
	Taizhou	1.085	6.5	0.574	0.07	3.5	0.526
Anhui	Hefei	3.372	10.5	0.634	0.33	7	0.583
	Wuhu	1.063	7.5	0.585	0.77	6	0.566
	Ma ’anshan	0.367	5	0.555	0.07	4.5	0.543
	Tongling	0.494	5.5	0.559	0.20	4	0.531
	Anqing	0.410	5	0.554	0.00	2.5	0.515
	Chuzhou	0.000	4	0.543	0.00	3	0.508
	Chizhou	0.200	3.5	0.537	1.56	5	0.542
	Xuancheng	0.000	3	0.531	0.57	4.5	0.539

From the perspective of tourist demand, Jiaxing, Zhoushan, Yancheng, and Taizhou (in Jiangsu province) are cities with development potential in the core circle of Shanghai and Nanjing. For example, the ancient towns of Wuzhen and Xitang in Jiaxing play an important role in the tourism circle of Shanghai with their ancient towns and the culture of water towns in the south of the Yangtze River Delta. Island tourism in Zhoushan also includes various tourism circles in the Yangtze River Delta region. The Chinese Milu Deer Park in Yancheng and the ancient town of Qintong in Taizhou (Jiangsu Province) are among the most popular tourist attractions in the Nanjing tourism circle. Scenic spots in Wenzhou, Taizhou (Zhejiang province), and Shaoshing are the main nodes attracting tourist flows in the Zhejiang tourism circle, while scenic spots in Hefei, Tongling, and Anqing are the main nodes attracting tourist flows in the Anhui tourism circle. These nodes also play an important role in attracting local tourists and need to be given more attention in the future planning of governments and tourism enterprises.

## Conclusion and discussion

This study used official news released by the Chinese government and the quotations for tour routes published by travel agencies as sources of data for the supply network. Travel notes from tourists’ blog communities about their travel experiences were used as sources of data for the demand network. This study analyzes the structural characteristics of regional tourism cooperation networks in the Yangtze River Delta from the perspective of supply and demand, and further discusses spatial differences within regional tourism cooperation supply and demand networks in the Yangtze River Delta.

The analysis results show that there are differences between the demand network of regional tourism cooperation formed by tourism flow and the supply network of regional tourism cooperation created by governments (tourism enterprises). As a result, the expected policies of and measures taken by governments and tourism enterprises may fail to achieve their desired effect. The Chinese governments (tourism enterprises) should make greater efforts to understand the preferences of tourists, make better use of web-based text data, and use joint marketing tools to promote the sustainable development of regional tourism cooperation in the Yangtze River Delta. In the future, the Chinese governments (tourism enterprises) need to adjust the relevant policies and measures in accordance with the following aspects. First, from the perspective of core–edge structure characteristics, in addition to supply and demand, both sides must focus more on Shanghai, Nanjing, Suzhou, and Hangzhou city, four core nodes. Wuxi, Nantong, Ningbo, and Hefei are also worthy of governments’ (tourism enterprises) attention to further cultivate core nodes. Second, from the perspective of the degree centrality index, governments (tourism enterprises) should pay more attention to the distribution function of four nodes, namely Wuxi, Changzhou, Nantong, and Ningbo; the diffusion function of Yangzhou, Zhenjiang, Hangzhou, Wenzhou, and Hefei; and the tourism resource development potential of Yancheng, Wenzhou, Shaoxing, Taizhou (in Zhejiang province), Hefei, and Tongling. Third, from the perspective of the betweenness centrality index, governments (tourism enterprises) should pay more attention to the transfer role of Wuxi, Changzhou, Nantong, and Jiaxing. Fourth, from the perspective of the spatial differences in the supply and demand networks, the nine cities of Suzhou, Zhenjiang, Yangzhou, Jinhua, Huzhou, Wuhu, Chizhou, Xuancheng, and Chuzhou play a weaker role as the central node in the demand network than the government (tourism enterprises) expected, indicating that the governments’ (tourism enterprises) policies have not achieved the expected effect. The 16 cities, including Wuxi, Changzhou, Hangzhou, Nantong, Ningbo, Jiaxing, Hefei, Yancheng, Wenzhou, Zhoushan, Taizhou (in Jiangsu province), Taizhou (in Zhejiang province), Ma’anshan, Shaoshing, Tongling, and Anqing, play a stronger role in the demand network than expected by the governments (tourism enterprises); therefore, governments and tourism enterprises need to pay more attention to them in the future.

Previous studies on tourist cities have focused on the analysis of their individual entities and the tourism products they provide [16, 24, 38–41]. The analysis of tourism cooperation in this study shows that the interpretation of individualism, essentialism, and atomism has shifted to a better understanding of relations, backgrounds, and systems [16]. Previously, scholars have examined the preconditions and policy framework of tourism cooperation in some specific regions [1, 4, 7, 9, 42], but there are few in-depth studies on tourism cooperation among cities in the Yangtze River Delta region of China. The present study intends to help fill this research gap by analyzing tourism cooperation from a supply and demand perspective through network analysis of official news released by the Chinese government and quotations for tour routes published by travel agencies. These data are then compared with the travel notes posted by tourists in China to provide a more accurate description about tourist cooperation in the Yangtze River Delta region of China.

### Implications

#### Theoretical implications

The theoretical contributions of this study are as follows: first, although the study of tourism cooperation network is very important, little attention has been paid to the understanding of destination cooperation network from the perspective of tourist demand [42]. This study explores the characteristics of tourism cooperation network structure among 26 cities in the Yangtze River Delta from the demand and supply sides, thus providing new knowledge. Second, this study is helpful to better understand how regional tourism cooperation network is constructed and to identify the internal mechanism of regional tourism cooperation network formation. This study not only helps to explore new areas of tourism research, but also helps to gain a deeper understanding of the effectiveness of government policies and measures taken by tourism enterprises. The results of this study complement and improve the previous qualitative research, and respond to a certain extent to the call for more quantitative research on tourism cooperation network [1, 3].

#### Practical implications

This study can provide important insights into the effective management of tourism destination governments and tourism enterprises. Tourism destination governments and tourism enterprises should optimize the tourism cooperation network structure of the Yangtze River Delta urban agglomeration based on the needs of tourists. For example, by identifying key participants in the tourism cooperation network, effective management by objectives should be formulated and implemented, which is particularly important for the improvement of the overall competitiveness of tourism destinations [19]. In addition, the location of destination in the tourism cooperation network affects the possibility of co-production. Managers should establish coordination mechanisms (e.g., alliances) between target participants based on the needs of tourists. High cooperation among cities in the Yangtze River Delta is not only conducive to attracting tourists, but also crucial to its sustainable development.

### Limitations and future research

It should be noted that no research method is perfect. Although the online text data of the network used in this study have many advantages, their sample representativeness, technical difficulties, and network population bias attributes also emerge as shortcomings, when compared with traditional research methods. In the future, we could consider collecting more extensive texts from more websites or new platforms to increase the representativeness of samples, or combine them with traditional research methods to corroborate each other and enhance the accuracy of research conclusions. Although the Yangtze River Delta region is regarded as a demonstration area for regional tourism cooperation, these research findings are not suitable for generalization to different regions of China or the world. In addition, structural holes and intermediaries in the supply and demand networks of tourism cooperation in the Yangtze River Delta need to be further explored. In fact, regional tourism cooperation can be multifaceted and multi-angled. It can be either formal or informal. Based on formal and informal relationships, this study interprets the structure of regional tourism cooperation networks from the aspects of supply and demand, which has rarely been examined in previous studies. This study is a descriptive pilot study designed to further refine some of the conceptual frameworks for regional tourism cooperation. Future research should turn to practical areas, aiming at analyzing the dynamics of tourism cooperation in the Yangtze River Delta, identifying the types of online information that the Chinese government and tourism enterprises may obtain, and exploring ways to make full use of tourist information to formulate policies or development strategies.

## Supporting information

S1 Data The demand network matrix. (XLSX)

S2 Data The supply network matrix. (XLSX)
